# Eggs as a Suitable Tool for Species Diagnosis of Causative Agents of Human Diphyllobothriosis (Cestoda)

**DOI:** 10.1371/journal.pntd.0004721

**Published:** 2016-05-25

**Authors:** Kateřina Leštinová, Miroslava Soldánová, Tomáš Scholz, Roman Kuchta

**Affiliations:** Institute of Parasitology, Biology Centre of the Czech Academy of Sciences & Faculty of Science, University of South Bohemia, České Budějovice, Czech Republic; Universidade Federal de Minas Gerais, BRAZIL

## Abstract

**Background:**

Tapeworms of the order Diphyllobothriidea are parasites of tetrapods and several species may infect man and cause neglected human disease called diphyllobothriosis. Identification of human-infecting diphyllobothriid cestodes is difficult because of their morphological uniformity, which concerns also their eggs in stool samples.

**Methods:**

In the present study, we analysed by far the largest dataset of more than 2,000 eggs of 8 species of diphyllobothriid cestodes that may infect humans, including the most frequent human parasites *Diphyllobothrium latum*, *D*. *nihonkaiense* and *Adenocephalus pacificus* (syn. *Diphyllobothrium pacificum*). Size (length, width and length/width ratio) and the surface of the egg shell from naturally and experimentally infected hosts were studied using light and scanning electron microscopy.

**Results:**

A high degree of intraspecific and host-related size variability has been detected, but combination of morphometrical and ultrastructural data made it possible to distinguish all of the studied species, including otherwise quite similar eggs of the 3 most common species infecting man, i.e. *D*. *latum*, *D*. *nihonkaiense* and *D*. *dendriticum*. The surface of all marine species is covered by numerous deep pits with species-specific density, whereas the surface of freshwater species is smooth or with isolated shallow hollows or wrinkles.

## Author Summary

More than 2,000 eggs of 8 species of diphyllobothriid cestodes infecting humans were compared. Combination of morphometrical and ultrastructural (surface morphology) data made it possible to distinguish all species.

## Introduction

Human infections with gastrointestinal helminths are usually diagnosed when their eggs are found in stool samples. Therefore, examination of stool and detection of parasite eggs is used as a non-invasive method for detection of infections [1]. Some of the helminth parasites are relatively easy to identify to the species level based on egg morphology, such as species of *Schistosoma* Weinland, 1858 (blood flukes) [1]. In contrast, eggs of diphyllobothriid tapeworms including human-infecting species are highly variable, their size may overlap between species and is also influenced by individual definitive hosts and their size. This makes reliable species diagnosis of causative agents of human diphyllobothriosis in stool samples difficult or even impossible [2].

Diphyllobothriosis and closely related diplogonoporosis are fish-borne zoonoses that are neglected, but widely distributed throughout the world; human infections with some of diphyllobothriids seem to have emerged recently [2,3,4,5]. Causative agents of these diseases are tapeworms of the genera *Adenocephalus* Nybelin, 1931, *Diphyllobothrium* Cobbold, 1858 and *Diplogonoporus* Lönnberg, 1892. Out of 16 species of diphyllobothriids reported from man, only the following 4 are common parasites of man, namely *Diphyllobothrium latum* (Linnaeus, 1758) with circumboreal distribution (with few cases reported also from Chile), *D*. *nihonkaiense* Yamane, Kamo, Bylund and Wikgren, 1986 in the northern Pacific region, *Adenocephalus pacificus* Nybelin, 1931 endemic in the southern Pacific, and *Diphyllobothrium dendriticum* (Nitzsch, 1824) with arctic distribution [2,3,4]. These cestodes, commonly called broad or fish tapeworms, produce up to 36,000 eggs per day [6,7]. Therefore, diagnosis of diphyllobothriosis is based mainly on findings of thick-shelled, unembryonated eggs with an operculum on the narrower end and a knob on the opposite site in stool samples [2].

The morphometry of eggs has been commonly used for species identification of diphyllobothriids in stool samples from naturally infected hosts including man and also experimentally infected unspecific hosts such as golden hamsters (*Mesocricetus auratus*) [8,9,10,11,12]. However, most authors used only ranges (minimum and maximum) of egg size in descriptions, which overlap among most of the species. Another limitation of previous studies represents a low number of measured eggs and the fact that only three species, *D*. *latum*, *D*. *dendriticum* and *D*. *nihonkaiense*, were studied in more detail.

Surface structures on the eggs and some other characters such as shell thickness, colour, diameter of an operculum, presence of an apical knob, or size of embryonic hooks were considered by previous authors, but none of them appeared suitable for species discrimination [13,14,15,16].

In the present study, we analysed so far the largest dataset of diphyllobothriid eggs from naturally and experimentally infected specific hosts, thus significantly improving upon previous studies on egg morphometry. The principal aim was to test whether combination of morphometrical and morphological features by light and/or scanning electron microscopy can make it possible to distinguish reliably the eggs of as many as 8 species of broad tapeworms reported from man.

## Materials and Methods

### Material studied

Gravid tapeworms and/or positive stool samples were obtained from naturally and experimentally infected specific hosts belonging to 19 species, including man; most samples were obtained from museum collections (see Table 1). They were identified using identification keys based on morphology and mostly also by genotyping (sequencing the *cox*1 gene) [17]. Morphometrical variability was studied in 62 samples of a total of 2,082 eggs of 8 species (Table 1); these samples were fixed in 70% ethanol, 4% formaldehyde solution or, in few cases, measured alive in the water. Measurements were taken using Olympus BX51 microscope with QuickPHOTO MICRO 2.3 program. In every sample, at least 25 intact eggs were measured to get representative dataset [18]. Standard deviation (SD), mean and length to width ratio (LWR) were counted using Microsoft Excel software. Summary data for egg measurements (in micrometres) pooled across all hosts for 3 morphometrical parameters (i.e. length, width and LRW; hereinafter referred to as ‘size’) are given in Table 2.

**Table 1:** Summary data for measurements and morphology of diphyllobothriidean egg samples from naturally and experimentally infected hosts.

Species	Definitive host	Locality	No.^1^	Range (mean) [μm]	No.^2^	Pits^3^	Code of samples^4^
*Adenocephalus*	*Arctocephalus pusillus*	Australia	26	46–54 × 36–42 (50 × 39)	3	–	IPCAS-AU 11
*pacificus*	*Arctophoca australis*	Argentina	25	49–54 × 39–43 (53 × 41)	5	80–87	IPCAS-ARG 28
	*Callorhinus ursinus*	USA, Alaska	25	50–62 × 40–48 (55 × 44)	2	46	IPCAS-SAM 0–17
			25	49–57 × 39–45 (54 × 43)	1	66	IPCAS-SAM 6–51
			25	51–60 × 39–47 (55 × 43)		–	IPCAS-SAM 7–64
	*Canis mesomelas*	South Africa	25	54–59 × 40–45 (57 × 42)	3	72–124	BMNH-1988.5.13.1–28
	*Homo sapiens*	Peru	25	43–51 × 35–42 (48 × 40)	5	34–71	IPCAS-TS 05/16
			25	47–55 × 37–44 (51 × 41)	7	–	IPCAS-TS 06/27
	*Neophoca cinerea*	Australia	24	41–57 × 40–48 (52 × 43)	4	79	IPCAS-AU 10
* *	*Otaria flavescens*	Peru	25	52–57 × 37–44 (55 × 41)	6	31–73	IPCAS-Peru 9
*Diphyllobothrium*	*Neomonachus*	USA, Hawaii	75	41–56 × 33–45 (47 × 39)	3	151–169	IPCAS-KEI 8–3
cf. *cameroni*	*schauinslandi*		25	45–52 × 36–41 (49 × 39)	6	99–127	USNPC-26205
*Diphyllobothrium*	*Canis familiaris*	Greenland	25	60–67 × 40–45 (63 × 42)	4	189–226	NHMD-DAN 26
*cordatum*			25	64–69 × 40–43 (67 × 42)	5	163–177	NHMD-DAN 28A
			25	71–79 × 45–53 (75 × 49)	4	98–150	NHMD-DAN 28B
	* *		25	61–69 × 40–44 (65 × 43)	3	89–113	NHMD-DAN 29
	*Erignathus barbatus*	Greenland	25	67–76 × 46–50 (72 × 48)	–	–	ZMUO-6
			25	70–78 × 46–50 (74 × 49)	3	103–163	NHMD-DAN 13A
			25	72–78 × 44–49 (76 × 47)	–	–	NHMD-DAN 13B
		USA, Alaska	50	65–78 × 43–54 (71 × 48)	1	172–194	IPCAS-PBI 429
	*Odobenus rosmarus*	Greenland	25	70–76 × 41–48 (73 × 44)	3	179–244	NHMD-DAN 2
		Russia, Bering Sea	25	66–77 × 47–53 (71 × 50)	3	140–177	IPCAS-TS 05/47
	* *	Russia, Chukotka	25	65–75 × 46–52 (70 × 49)	5	187–211	IPCAS-TS 04/27
*Diphyllobothrium*	*Canis familiaris*	Russia	25	57–63 × 38–45 (60 × 40)	5	0	IPCAS-TS 04/39
*dendriticum*	*Larus hyperboreus*	USA, Kansas	25	53–66 × 38–43 (59 × 40)	1	0	IPCAS-KS-6
* *	*Mesocricetus auratus*^5^	Norway	74	49–64 × 37–49 (58 × 41)	4	0	ZMUO-437
*Diphyllobothrium*	*Monachus monachus*	Italy	25	59–63 × 44–47 (61 × 45)	6	97–109	NHMD-DAN 15
*hians*	* *		50	45–65 × 35–50 (59 × 46)	–	–	IPCAS-ITA 140
*Diphyllobothrium*	*Canis familiaris*	Russia	25	61–68 × 44–48 (65 × 45)	7	0	IPCAS-S 04/17
*latum*	*Canis lupus*	Switzerland	25	60–65 × 42–46 (63 × 44)	4	0	MHNG-56104
	*Homo sapiens*	Australia	25	66–73 × 49–53 (70 × 50)	3	0	QM-GL 12348
		Chile	25	65–73 × 48–53 (70 × 51)	4	0	IPCAS-Chile 1a 2012
			25	67–74 × 46–52 (71 × 50)	3	0	IPCAS-Chile 1b 2014
		Czech Republic	25	61–66 × 44–48 (64 × 46)	5	0	IPCAS-CZ 78
		Italy	274	60–81 × 43–57 (70 × 50)	–	–	IPCAS-CZ 79a
			141	62–76 × 47–58 (69 × 51)	13	0	IPCAS-CZ 79b
		Norway	25	60–66 × 43–49 (63 × 47)	5	0	ZMUO-C 1508
			25	62–71 × 44–51 (68 × 48)	–	–	ZMUO-C 1513
			25	60–65 × 40–46 (62 × 44)	–	–	ZMUO-C 1515
			25	61–68 × 43–48 (65 × 45)	3	0	ZMUO-C 1517
		Russia	25	62–70 × 45–50 (65 × 47)	–	–	IPCAS-CZ 86
	* *		25	64–69 × 47–51 (66 × 48)	–	–	IPCAS-RUS 106
		Switzerland	25	68–76 × 49–54 (72 × 51)	4	0	MHNG-38373
	*Mesocricetus auratus*^5^	Italy	50	57–68 x 43–49 (63 x 46)	–	–	IPCAS CZ 79 Ha
		Norway	25	57–62 x 40–43 (59 x 41)	–	–	ZMUO 10
			25	51–58 x 39–44 (55 x 42)	–	–	ZMUO 2
			23	54–65 x 39–45 (59 x 42)	7	0	ZMUO 7A
		Switzerland	25	61–69 x 42–50 (65 x 46)	4	0	MHNG-17860
* *	*Ursus maritimus*	Austria (ZOO)	25	66–74 × 46–52 (70 × 49)	3	0	NMW-20045
*Diphyllobothrium*	*Homo sapiens*	Japan	25	59–64 × 40–43 (62 × 42)	3	0	IPCAS-2010-67
*nihonkaiense*			25	59–66 × 41–44 (62 × 42)	7	0	IPCAS-2010-67-E
			25	60–66 × 39–45 (63 × 42)	6	0	IPCAS-2013-16
			25	59–66 × 38–44 (62 × 41)	–	–	IPCAS-2013-52
			25	58–66 × 41–44 (61 × 43)	–	–	IPCAS-2014-01
			25	60–67 × 40–48 (63 × 44)	–	–	IPCAS-2014-63
			25	63–68 × 40–48 (65 × 44)	2	0	IPCAS-Dn 2014–1
			25	61–69 × 40–48 (65 × 44)	–	–	IPCAS-Dn 2014–2E
			25	60–63 × 39–43 (61 × 40)	2	0	IPCAS-Dn 2014–2S
* *	* *		21	55–66 × 44–47 (61 × 46)	3	0	IPCAS-Dn1
*Diphyllobothrium*	*Lagenorhynchus acutus*	USA, Mississippi	25	53–64 × 41–48 (60 × 46)	4	78–92	IPCAS-TS 09/101
*stemmacephalum*	*Phocoena*	Ukraine, Black Sea	25	63–70 × 44–47 (67 × 46)	–	–	GenBank-DQ768191
* *	*Tursiops truncatus*	USA, Massachusetts	24	64–70 × 40–50 (66 × 47)	–	–	IPCAS-USA 21
TOTAL:	62 samples (56 analyzed)		2,082 (1,860 analyzed)		184		

^1^Number of eggs measured

^2^Number of eggs observed by scanning electron microscopy

^3^Number of pits per 100 μm^2^

^4^Deposited in the Natural History Museum, London, UK (BMNH), Institute of Parasitology, CAS, České Budějovice, Czech Republic (IPCAS), Muséum d’Histoire Naturelle, Geneva, Switzerland (MHNG), Statens Naturhistoriske Museum, Copenhagen, Denmark (NHMD), Naturhistorisches Museum, Vienna, Austria (NMW), Queensland Museum, Australia (QM), United States National Parasite Collection, Beltsville, Maryland, USA (USNPC), Zoological Museum, University of Oslo, Norway (ZMUO)

^5^Experimentally infected atypical host–golden hamster; not used in statistical analysis.

**Table 2:** Descriptive statistics for measurements of eggs of 8 diphyllobothriidean species studied from natural (samples from same hosts are pooled). Range and means are given in micrometers for the 3 parameters subjected to morphometrical studies. Details for individual samples are listed in Table 1. Measurements are in micrometers (μm).

Species^1^	Hosts^2^	No.^3^	Length^4^	Width^4^	Length width ratio^4^
Apa	1, 3, 6–10	250 (13)	41–62 (53 ± 3.32)	35–48 (42 ± 2.24)	0.953–1.475 (1.272 ± 0.079)
Dca	11	100 (2)	41–56 (48 ± 2.95)	33–45 (39 ± 2.17)	1.079–1.441 (1.218 ± 0.071)
Dco	2, 12, 13	300 (11)	60–79 (71 ± 4.22)	40–54 (47 ± 3.28)	1.259–1.727 (1.519 ± 0.088)
Dde	2, 18	50 (2)	53–66 (60 ± 2.44)	38–45 (40 ± 1.75)	1.325–1.632 (1.480 ± 0.067)
Dhi	14	75 (2)	45–65 (60 ± 3.42)	35–50 (46 ± 2.40)	1.143–1.444 (1.309 ± 0.062)
Dla	1, 2, 4, 5	765 (16)	60–81 (68 ± 3.54)	40–58 (49 ± 2.89)	1.172–1.600 (1.389 ± 0.066)
Dni	1	246 (11)	55–69 (63 ± 2.29)	38–48 (43 ± 2.13)	1.217–1.737 (1.463 ± 0.082)
Dst	15–17	74 (3)	53–70 (64 ± 3.75)	41–50 (46 ± 1.56)	1.191–1.556 (1.383 ± 0.078)

^1^Apa *Adenocephalus pacificus*, Dca *Diphyllobothrium* cf. *cameroni*, Dco *D*. *cordatum*, Dde *D*. *dendriticum*, Dhi *D*. *hians*, Dla *D*. *latum*, Dni *D*. *nihonkaiense*, Dst *D*. *stemmacephalum*

^2^1. *Homo sapiens*, 2. *Canis familiaris*, 3. *C*. *mesomelas*, 4. *C*. *lupus*, 5. *Ursus maritimus*, 6. *Otaria flavescens*, 7. *Arctocephalus pusillus*, 8. *Arctophoca australis*, 9. *Callorhinus ursinus*, 10. *Neophoca cinerea*, 11. *Neomonachus schauinslandi*, 12. *Erignathus barbatus*, 13. *Odobenus rosmarus*, 14. *Monachus monachus*, 15. *Lagenorhynchus acutus*, 16. *Tursiops truncatus*, 17. *Phocoena phocoena*, 18. *Larus hyperboreus*

^3^Number of measured eggs (number of samples)

^4^Range (minimum and maximum); mean ± SD in parentheses.

A series of univariate comparisons was performed by separate one-way analyses of variance (One-way ANOVA’s) for sets of analyses (A1–A3 –see Table 3) in which different numbers of specimens and parasite species were used to assess: (A1) differences in egg sizes (i.e. 3 morphometrical features: length, width and LRW) among all 8 diphyllobothriid species; (A2) differences in egg sizes among the 3 commonest human species, namely *A*. *pacificus*, *D*. *latum* and *D*. *nihonkaiense*; and (A3) intraspecific variability in egg sizes of the species from minimally 3 different hosts were available: (A3a) *A*. *pacificus*, (A3b) *D*. *latum*, and (A3c) *D*. *cordatum*. Samples from hamster were not included because it is an atypical host and their eggs are considerably variable compared to specific hosts (Table 1). Accordingly, 56 samples of a total of 1,860 eggs were entered to statistical analyses (Tables 1 and 3). All data were log-transformed (log_10_), but absolute values of egg sizes were used in graphs for better illustration. *Post-hoc* Tukey HSD tests were performed where appropriate to detect differences in particular morphometrical features among species pairs. All analyses were carried out using Statistica 7.0 software package (StatSoft Inc., Tulsa, OK, USA) with significance levels set at 0.05.

**Table 3:** Summary data for egg samples of diphyllobothriidean cestodes from naturally infected hosts used in analysis of variance (one-way ANOVA) statistics assessing interspecific and intraspecific variation in sizes (length, width and length/width ratio). See Table 1 for data on hosts.

Analysis	No. of species	No.^a^	MP^b^	Df^c^	F^d^	P^e^
A1	All 8 species^f^	1,860 (56)	Length	7	1,154	< 10^−4^
			Width	7	435	< 10^−4^
			LWR^a^	7	358	< 10^−4^
A2	3 –*A*. *pacificus*	986 (24)	Length	2	1,228	< 10^−4^
	*D*. *latum*		Width	2	853	< 10^−4^
	*D*. *nihonkaiense*		LWR	2	295	< 10^−4^
A3a	1 –*A*. *pacificus*	250 (10)	Length	6	41	< 10^−4^
			Width	6	30	< 10^−4^
			LWR	6	19	< 10^−4^
A3b	1 –*D*. *latum*	765 (16)	Length	3	32	< 10^−4^
			Width	3	61	< 10^−4^
			LWR	3	12	< 10^−4^
A3c	1 –*D*. *cordatum*	300 (11)	Length	2	65	< 10^−4^
			Width	2	84	< 10^−4^
			LWR	2	6	< 10^−4^

^a^Number of measured eggs (number of samples)

^b^Morphometrical parameter

^c^Degrees of freedom

^d^F-test values

^e^Level of significance

^f^*Adenocephalus pacificus*, *Diphyllobothrium* cf. *cameroni*, *D*. *cordatum*, *D*. *dendriticum*, *D*. *hians*, *D*. *latum*, *D*. *nihonkaiense*, *D*. *stemmacephalum*.

Additionally, 184 eggs of 44 samples of all 8 studied species were studied using scanning electron microscopy (SEM) (Table 1). Proglottids with eggs were prepared as outlined by Kuchta and Caira [19]. Briefly, after drying the eggs were liberated by dissecting needles from gravid proglottids during mounting on aluminium stubs using a double-sided tape. Samples were examined by JEOL JSM-7401F scanning electron microscope. Surface structures were observed at magnification ×10,000 and the density of the pits was re-counted for 100 μm^2^.

### Ethics statement

Eggs of diphyllobothriidean cestodes from human samples were obtained from the following museum collections (in most cases, proglottids found in stool samples were fixed and deposited in the collections; the eggs were obtained as described above): Institute of Parasitology, CAS, České Budějovice, Czech Republic (IPCAS), Muséum d’Histoire Naturelle, Geneva, Switzerland (MHNG), Queensland Museum, Australia (QM), and Zoological Museum, University of Oslo, Norway (ZMUO). In addition, samples of eggs from non-human hosts were obtained from museum material deposited in: Natural History Museum, London, UK (BMNH), IPCAS, MHNG, Statens Naturhistoriske Museum, Copenhagen, Denmark (NHMD), Naturhistorisches Museum, Vienna, Austria (NMW), United States National Parasite Collection, Beltsville, Maryland, USA (USNPC), ZMUO (Table 1).

## Results

### Morphometry of the eggs

**Fig 1:**
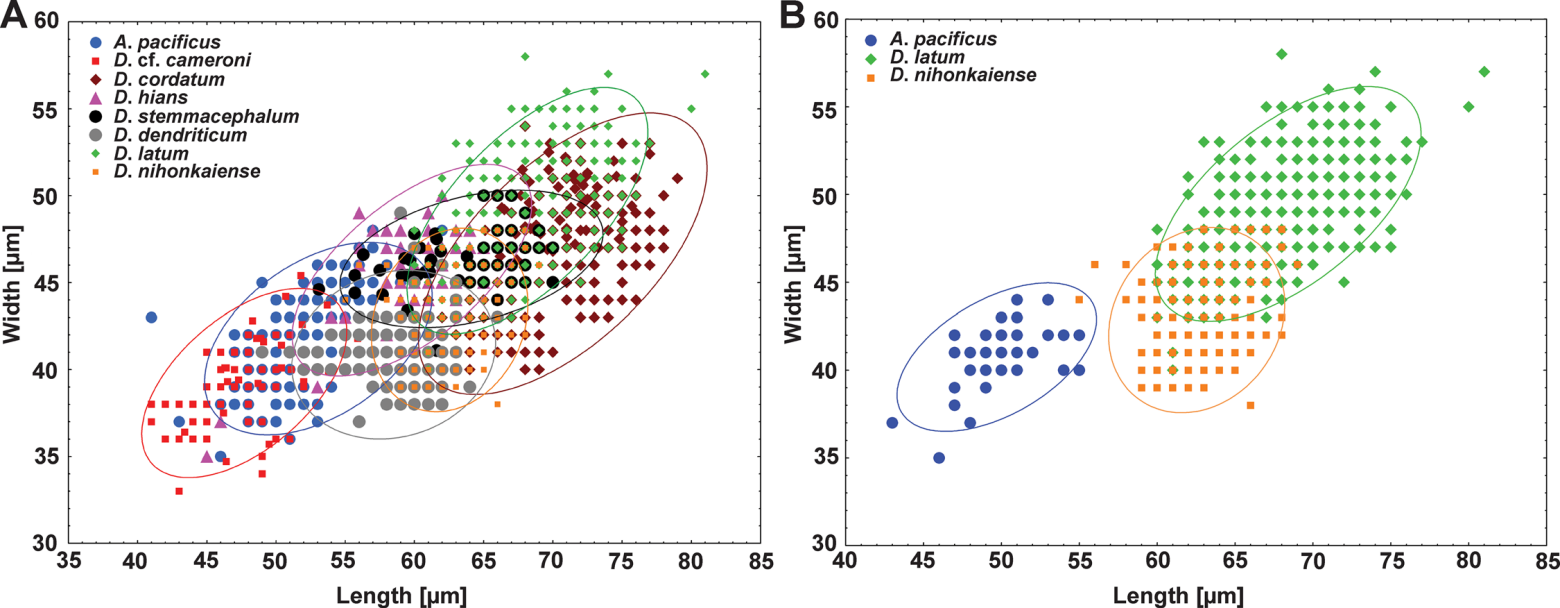
The scatterplot displaying the positive association between the length and width of eggs of diphyllobothriid cestodes. *A*, Based on 56 samples of 8 species from various definitive hosts. *B*, Based on 3 commonest species infecting humans for 25 samples exclusively from man. Ellipses represent 95% confidence intervals about the means indicating greater correlation between length and width in a given species. Details for individual samples are listed in Table 1.

**Fig 2:**
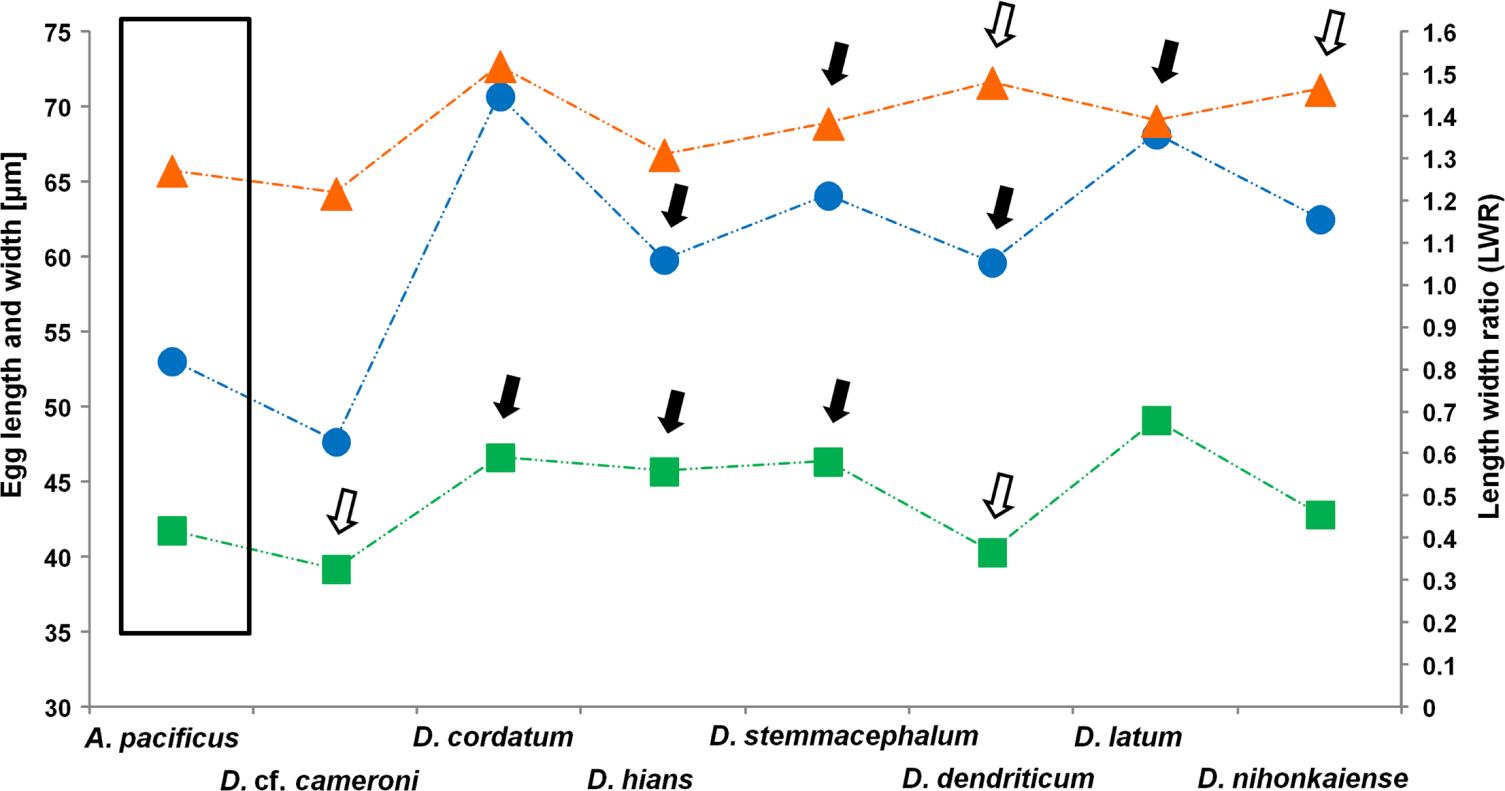
Comparison of the means of 3 morphometrical parameters (length in circles, width in squares and length to width ratio in triangles) measured on eggs of 8 species of diphyllobothriid cestodes. Black and white arrows indicate pairs/groups of species for which statistical difference of particular parameter was not detected, thus showing similar values for these features. Species differing in all tested parameters is marked with a square (*A*. *pacificus*) and can be easily distinguished from the remaining species.

Morphometrical analysis of all eggs has shown a great size variability of most species studied (Fig 1). The results of analysis A1 showed significant differences in egg sizes among all 8 species (P < 10^−4^ for all 3 morphometrical features tested; Fig 2; Table 3). The *post-hoc* test showed significant differences between most of the species except *D*. *dendriticum* and *D*. *hians*, for which egg measurements overlap in length (P > 0.05) demonstrating no apparent difference in this parameter (Fig 2). *Diphyllobothrium cordatum* has the largest eggs (mean length 71) followed by *D*. *latum* (68) and *D*. *stemmacephalum* (64), whereas *D*. cf. *cameroni* and *A*. *pacificus* have the smallest eggs (48 and 53, respectively; Fig s 1 and 2; Tables 1 and 2).

Concerning egg width, measurements divided all species into 2 species groups. The first group includes *D*. *cordatum*, *D*. *hians* and *D*. *stemmacephalum*, and second one *D*. *dendriticum* and *D*. cf. *cameroni*; they did not differ from each other (P > 0.05) (Fig 2; depicted by black and white arrows, respectively). The widest eggs were found in *D*. *latum* (mean width 49), followed by *D*. *cordatum* (47), whereas *D*. *cameroni* and *D*. *dendriticum* have the narrowest eggs (39 and 40, respectively; Table 2).

The *post-hoc* test revealed that the eggs of *D*. cf. *cameroni* and *A*. *pacificus* are rounder than those of the 6 remaining species (mean LWR 1.218 and 1.272, respectively; Table 2). This morphometrical parameter significantly differs among all but 2 pairs of species, *D*. *latum* and *D*. *stemmacephalum*, and *D*. *dendriticum* and *D*. *nihonkaiense* (P > 0.05), which cannot be distinguished based on this character (Fig 2; depicted by black and white arrows, respectively).

One of the 8 species, *A*. *pacificus*, can be distinguished from the 7 remaining species by all 3 morphometrical features (length, width and LWR; Fig 2; Table 3). The remaining species form 5 groups in which a given species overlaps in one particular parameter, but significantly differs in the other two, thus making them distinguishable among each other as well. For example, *D*. *hians* and *D*. *dendriticum* create the first group by overlapping measurements in length but are different in width and LWR index (Fig 2; Table 2).

Univariate comparisons (analysis A2) of the eggs among the 3 commonest human-infecting species, i.e. *A*. *pacificus*, *D*. *latum* and *D*. *nihonkaiense*, showed significant differences in all 3 parameters tested (Table 3). However, despite these differences, the range of measurements of *D*. *latum* and *D*. *nihonkaiense* slightly overlapped in all 3 characters (Figs 1 and 2; Table 2). The eggs of *A*. *pacificus* are smallest (the lowest mean length and width) as well as most rounded (Figs 1 and 2; Table 2). In contrast, *D*. *latum* possesses the largest eggs and the eggs of *D*. *nihonkaiense* have the highest LWR, i.e. more elongate eggs (Table 2).

**Fig 3:**
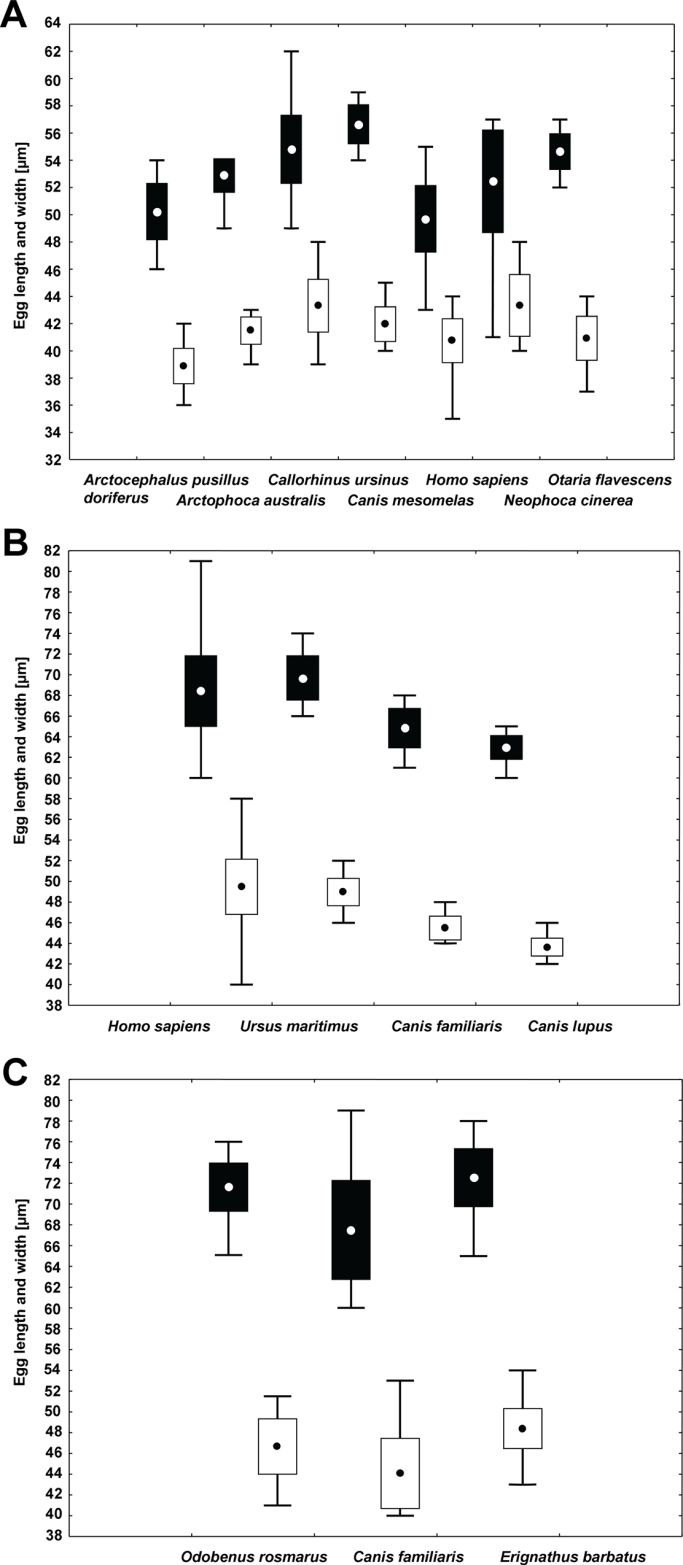
Intraspecific variability of egg sizes of selected diphyllobothriid species. *A*, *Adenocephalus pacificus* from 7 host species. *B*, *Diphyllobothrium latum* from 4 host species. *C*, *Diphyllobothrium cordatum* from 3 host species. Data represent mean values for length (in black) and width (in white). Whiskers indicate maximum and minimum values of measured eggs, boxes standard deviation of the mean and circles inside of boxes the mean value.

The eggs of *A*. *pacificus*, *D*. *latum* and *D*. *cordatum* from various hosts (analyses A3a–A3c) differed significantly in all 3 morphometrical parameters (Table 3). The eggs of *A*. *pacificus* from a jackal, *Canis mesomelas*, were on average longest compared to conspecific eggs from 6 other hosts and differed in width from 2 hosts only (Fig 3; Table 1). The eggs of *A*. *pacificus* from humans and from fur seal, *Arctocephalus pusillus*, were shortest on average. The widest eggs of *A*. *pacificus* were found in sea lion, *Neophoca cinerea*, and fur seal, *Callorhinus ursinus* (Fig 3; Table 1). The most elongate eggs come from *Neophoca cinerea* and the most rounded from a jackal (Fig 3, Table 1).

Significant differences were detected in all morphometrical features of the eggs of *D*. *latum* from different hosts (analysis A3b; Table 3). Eggs from humans and polar bear were longest and widest compared to the eggs from dog and wolf (Fig 3; Table 1). Eggs from human stool samples were also most elongate (Table 1).

The eggs of *D*. *cordatum* from 3 different hosts also differed significantly from each other in all parameters (P < 0.001; Fig 3; Table 3). The eggs from dogs were smaller compared to those from seals (Fig 3) whereas most elongate eggs were from *Erignathus barbatus* (Table 1).

### Egg surface

**Fig 4:**
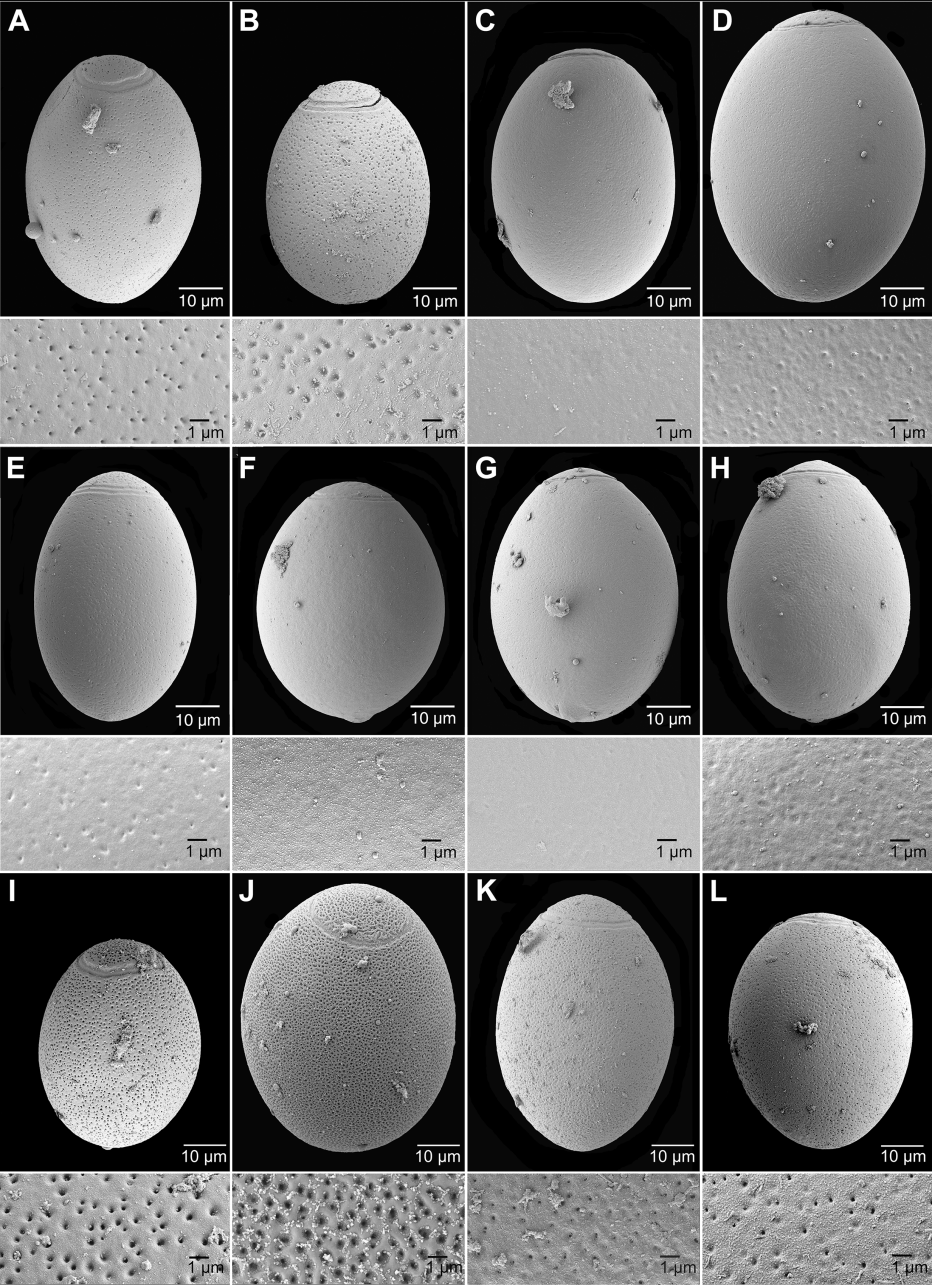
Scanning electron micrographs of eggs of marine (m) and freshwater /anadromous (f) diphyllobothriid cestodes and their surface. *A*–*B*, *Adenocephalus pacificus* (m) ex *Canis mesomelas* (*A*) and *Arctocephalus australis* (*B*). *C*–*D*, *Diphyllobothrium latum* (f) ex *C*. *lupus* (*C*) and *Homo sapiens* (*D*). *E*, *D*. *dendriticum* (f) ex *C*. *familiaris*. *F*–*H*, *D*. *nihonkaiense* (f) ex *H*. *sapiens*. *I*, *D*. cf. *cameroni* (m) ex *Neomonachus schauinslandi*. *J*, *D*. *cordatum* (m) ex *Erignathus barbatus*. *K*, *D*. *hians* (m) ex *Monachus monachus*. *L*, *D*. *stemmacephalum* (m) ex *Lagenorhynchus acutus*. Figures are showing eggs and their surface in the same magnification 10.000×.

**Fig 5:**
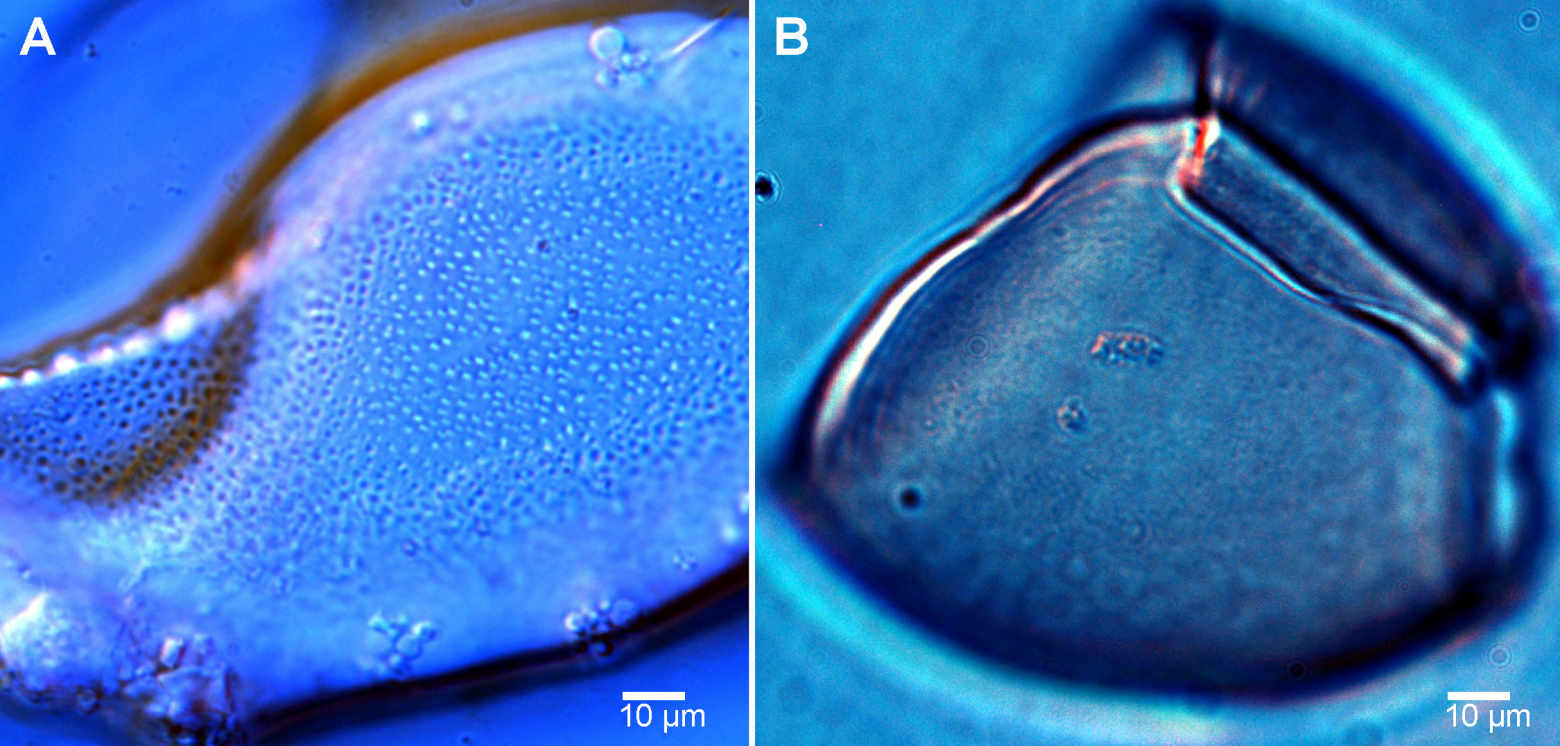
Photomicrographs of the surface of diphyllobothriid eggs observed by light microscopy. *A*, marine species *Diphyllobothrium cordatum* ex *Erignathus barbatus* covered by numerous deep pits. *B*, anadromous species *Diphyllobothrium nihonkaiense* ex *Homo sapiens* smooth or with isolated shallow hollows or wrinkles.

Eggs of all marine species (*Adenocephalus pacificus*, *D*. cf. *cameroni*, *D*. *cordatum*, *D*. *hians* and *D*. *stemmacephalum*) differ conspicuously from those of freshwater/anadromous species (*D*. *dendriticum*, *D*. *latum* and *D*. *nihonkainese*) in the presence of numerous deep pits on their surface observed using SEM or light microscope with a high magnification and strong pressing coverslip with egg samples (Figs 4 and 5) [14,20]. Density of pits of marine species differed between species, from 34 pits per 100 μm^2^ in *A*. *pacificus* to as many as 207 in *D*. *cordatum* (Fig 4; Table 1). This characteristic may help as an additional feature to distinguish species groups with similar egg sizes, such as *D*. *hians* from those of *D*. *stemmacephalum* (Figs 2 and 4; Tables 1 and 2). The eggs of the remaining freshwater/anadromous species are indistinguishable from each other based on their surface, because it is smooth or occasionally covered with a few shallow hollows or wrinkles (Fig 4; Table 1).

To facilitate species diagnosis of eggs in stool (clinical) samples, a simplified key for identification of the eggs of 8 human-infecting species of diphyllobothriid cestodes based on their length and width is provided. The mean value represent means between the species from each compared groups with closest values, i.e. the smallest value in the larger group and largest values in the smaller group and species of the genus *Diplogonoporus* are not considered (see Discussion). The present key does not include data on pits observed on the eggs from jackal as an unspecific host (see Table 1).

1a Mean length less than 56.5 μm ……..…. . . .. . . ..……. . . .. . . .. . . .. . . .. . . .. . . .. . .. . . 2
1b Mean length more than 56.5 μm ……………….………. . . .. . .. . ..……. . . 3
2a Mean length 50.5 μm; more than 98 pits per 100 μm^2^…. . . .. . . .. . . .. . . .. . .. . . *D*. cf. *cameroni*
2b Mean length 50.5 μm; less than 85 pits per 100 μm^2^……. . . .. . . .. . .. . ... . . . *A*. *pacificus*
3a Mean width less than 44.5 μm …. . . .. . . .. . . .. . . .. . . .. . . .. . . .. . . .. . . .. . . .. . . .. . . .. . . .. . .. . . 4
3b Mean width more than 44.5 μm …………….……. . . .. . . .. . . .. . . .. . . .…. . .. . .. 5
4a Mean width less than 42 μm; mean length less than 61.5 μm …. . . .. . .. . . *D*. *dendriticum*
4b Mean width more than 42 μm; mean length more than 61.5 μm. . . .. . .. . .. *D*. *nihonkaiense*
5a Mean length less than 69.5 μm; mean LWR less than 1.50. . . .. . . .. . . .. . .. . . 6
5b Mean length more than 69.5 μm; mean LWR more than 1.50. . . .. . . .. . . .. *D*. *cordatum*
6a Mean length less than 66 μm; mean width less than 47.5 μm. . . .. . . .. . .. . . 7
6b Mean length more than 66 μm; mean width more than 47.5 μm. . . .. . . .. *D*. *latum*
7a Mean length less than 62 μm; mean LWR less than 1.35. . . .. . . .. . . .. . . .. . . . *D*. *hians*
7b Mean length more than 62 μm; mean LWR more than 1.35. . . .. . . .. . . .. . . .. *D*. *stemmacephalum*

## Discussion

Human diphyllobothriosis is not a life-threating disease and symptoms are usually mild despite the large size of tapeworms (up to 20 m). However, the number of human case does not decline, reaching about 20 million cases globally [21]. In contrast, several species seem to (re-)emerge or neglected even in the most developed countries [2,3,22]. For clinicians, species-specific identification of eggs in stool samples is not necessary because clinical symptoms are similar in all human-infecting species [2]. However, proper species identification is crucial from the epidemiological point of view, i.e. to detect sources of human infection, actual distribution of potential human-infecting species and way of transmission of plerocercoids to humans [2].

The present study represents the most complex statistical analysis based on the so far largest dataset for diphyllobothriid eggs. The main novelty of this study is combination of morphometrical and ultrastructural characteristics of diphyllobothriid eggs which enabled us to distinguish individual human-infecting species from each other. Species-specific identification of eggs is thus possible despite a rather high degree of their morphometrical and morphological variability in most of the studied species observed in the present and previous studies (see below).

Previously, the most extensive study of diphyllobothriid eggs was carried out by Hilliard [14] who studied 8 species from naturally and experimentally infected hosts in Alaska and by Andersen and Halvorsen [10] who compared the 3 commonest freshwater species in Europe, *D*. *ditremum*, *D*. *dendriticum* and *D*. *latum*, from a variety of definitive hosts. Despite some differences, especially in the size of the eggs of *D*. *cordatum* from bearded seal, *E*. *barbatus* [14], the present data correspond to those provided by previous authors [10,14,23,24].

Additional study based on 7 species from human clinical samples studied by Maejima [25] focused mainly on eggs of *D*. *latum*, *D*. *nihonkaiense*, *A*. *pacificus* and *D*. *stemmacephalum* (reported as *D*. *yonagoense*). These data correspond more or less with those of the present study, even though values reported for the 2 former species were somewhat lower than herein.

The eggs of *D*. *latum* have been studied most intensively, especially those from man; a high degree of size variability (55–81 × 40–59) has been detected [10,26; present study]. However, some of these records may in fact have included other species such as *D*. *nihonkaiense* in Far East Asia and *D*. *dendriticum* in temperate zones [2,12,27].

The detailed study comparing the 3 commonest freshwater species in Europe, *D*. *ditremum*, *D*. *dendriticum* and *D*. *latum*, from different definitive hosts was carried out by Andersen and Halvorsen [10]. They observed great variation in mean egg size among different worms belonging to the same species as well as among different species. It was concluded that size of the eggs also depends on the size and type of definitive hosts [10,11,28].

The intraspecific variability based on host species was compared in 3 species in the present study. Significant differences were found in all 3 species, but their ranges overlapped in almost all host groups (Fig 3). In general, there is tendency of tapeworms from larger specific hosts to have larger eggs, but that scenario is not confirmed by all studies. For example, the present study shows that eggs of *A*. *pacificus* from man (atypical host) are significantly smaller than those from pinnipeds (specific hosts) but, surprisingly, the longest eggs are those from jackal, which is an atypical host (Fig 3; Table 1) [17]. Moreover, some previous studies did not find significant differences between the size of eggs of *A*. *pacificus* from man and those from fur seals, even though the eggs from man were slightly smaller and the surface was covered with more pits [11,28]. Eggs from atypical experimental hosts such as golden hamster may be larger than those from typical hosts as observed in *D*. *dendriticum* or *D*. *latum* [10; Table 1].

The present study of egg surface confirmed differences between freshwater and marine species reported by previous authors [14,29,30,31]. The surface of the eggs of all marine species studied is covered with numerous deep pits, whereas freshwater species have a smooth surface or only a limited number of wrinkles or shallow pits (Fig 4). It is important to point out that only clean eggs should be used for SEM observations to avoid misinterpretation of artifacts as natural surface structures. The egg surface can be observed also by light microscope if eggs are strongly compressed under coverslip and observed (Fig 5) [14,20]. Future laboratory experiments with eggs of marine and freshwater species are necessary to provide an explanation this difference in surface structure. The eggs of diphyllobothriid cestodes are heavy and cannot float in the water; instead they sink after their shedding, but the presence/absence of numerous and deep pits on their surface seem to be related to different physical properties of fresh and sea water and certainly plays some, yet unknown role in facilitating parasite transmission [14,29].

Combination of morphometry and surface ultrastructure of the eggs appeared to be helpful in identification of 8 human-infecting diphyllobothriideans, including *D*. *latum*, *D*. *dendriticum* and *D*. *nihonkainse*, which are very similar in morphology and morphometry of their adults and eggs [2,12]. However, this is possible only if a sufficient number of the eggs (at minimum 25) is measured to avoid statistical error due to a high variability of eggs and to enable reliable species-specific identification [18].

The eggs of the common species *A*. *pacificus* can be identified most easily because they are smallest and their surface is covered, similarly as in other marine species, with numerous pits (Figs 2 and 4; Table 1). However, we are well aware that such a detailed morphometrical and morphological analysis that include preferentially scanning electron microscopy is not possible during routine diagnosis of the stool samples in clinical laboratories. Therefore, positive clinical samples, especially in non-endemic areas, should be always fixed first with ethanol and later identified by molecular methods, mainly by sequencing of the mitochondrial *cox*1 gene or by available multiplex PCR [2,31].

In clinical samples, eggs of other helminths of similar shape and size can be found. The most similar are those of another diphyllobothriideans normally infecting whales, *Diplogonoporus balaenopterae* Lönnberg, 1892, which causes human diplogonoporosis [2]. The eggs of this cestode from human samples are 57–80 long and 34–49 wide; their surface is covered with numerous pits (150–250 pits per 100 μm^2^) [14,20,29,30]. They can thus be confused with those of *Diphyllobothrium cordatum* and *D*. *hians* (see Table 2). However, the number of human cases of diplogonoporosis is quite low, around 200 worldwide [2].

To conclude, the present study provides evidence that combination of several characteristics assessed by statistical methods represents a useful tool to differentiate otherwise indistinguishable eggs of human-infecting broad fish tapeworms. Even though a detailed morphometrical and morphological (ultrastructural) characterisation of the diphyllobothriid eggs is not trivial, but relatively fast and cheap and could be used for routine diagnostics. Accurate identification of the species causing diphylloobothriosis is essential for understanding of the epidemiology and transmission of this neglected fish-borne human disease, which seems to (re-)emerged due to changing eating habit even in the most developed countries.

## References

[1] Ash LR, Orihel TC (2007) Atlas of Human Parasitology. Singapore: ASCP Press.

[2] Kuchta R, Scholz T, Brabec J, Narduzzi-Wicht B (2015) Chapter 17. *Diphyllobothrium*, *Diplogonoporus* and *Spirometra* In: Xiao L, Ryan U, Feng F, editors. Biology of Foodborne Parasites Section III Important Foodborne Helminths: CRC Press pp. 299–326.

[3] Kuchta R, Brabec J, Kubáčková P, Scholz T (2013) Tapeworm *Diphyllobothrium dendriticum* (Cestoda)–Neglected or emerging human parasite? PLoS Negl Trop Dis 7: e2535 doi: 10.1371/journal.pntd.0002535 24386497 10.1371/journal.pntd.0002535 PMC3873255

[4] Kuchta R, Serrano-Martinez ME, Scholz T (2015) Pacific broad tapeworm *Adenocephalus pacificus* as a causative agent of globally reemerging diphyllobothriosis. Emerg Infect Dis 21: 1697–1703. doi: 10.3201/ 26402440 10.3201/eid2110.150516 PMC4593442

[5] de Marval F, Gottstein B, Weber M, Wicht B (2013) Imported diphyllobothriasis in Switzerland: molecular methods to define a clinical case of *Diphyllobothrium* infection as *Diphyllobothrium dendriticum*. Eurosurveillance 18: 31–36. 23351654

[6] Nicholson D (1928) Fish tapeworm, intestinal infection in man: The infestation of fish in Manitoba lake. Can Med Assoc J 19: 25–33. PMC1709739 20316917

[7] Bonsdorff Bv (1977) Diphyllobothriasis in man London: Academic Press.

[8] Essex HE, Magath TB (1931) Comparison of the variability of ova of the broad fish tapeworm, *Diphyllobothrium latum*, from man and dogs: its bearing on the spread of infestation with this parasite. Am J Hyg 14: 698–704.

[9] Vergeer T (1936) The eggs and the coracidia of *Diphyllobothrium latum*. Papers of the Michigan Academy of Sciences, Arts and Letters 21: 715–727.

[10] Andersen KI, Halvorsen O (1978) Egg size and form as taxonomic criteria in *Diphyllobothrium*. Parasitology 76: 229–240. 652389 10.1017/s0031182000047818

[11] Tsuboi T, Torii M, Hirai K (1993) Light and scanning electron microscopy of *Diphyllobothrium pacificum* expelled from a man. Jpn J Parasitol 42: 422–428.

[12] Choi S, Cho J, Jung B-K, Kim D-G, Jeon S, et al (2015) *Diphyllobothrium nihonkaiense*: wide egg size variation in 32 molecularly confirmed adult specimens from Korea. Parasitol Res 114: 2129–2134. doi: 10.1007/s00436-015-4401-7 25758587 10.1007/s00436-015-4401-7

[13] Fraser PG (1960) The form of the larval hooks as a means of separating species of *Diphyllobothrium*. J Helminthol 34: 73–80.

[14] Hilliard DK (1960) Studies on the helminth fauna of Alaska. XXXVIII. The taxonomic significance of eggs and coracidia of some diphyllobothriid cestodes. J Parasitol 46: 703–715. 13714326

[15] Vik R (1964) The genus *Diphyllobothrium*. An example of the interdependence of systematics and experimental biology. Exp Parasitol 15: 361–380. 14201367 10.1016/0014-4894(64)90031-1

[16] Yazaki S (1982) Studies on three dimentional features of embryonic hooks in some species of diphyllobothriids, with special reference to taxonomic significance. Jpn J Parasitol 31: 435–446. (In Japanese.)

[17] Hernández-Orts JS, Scholz T, Brabec J, Kuzmina T, Kuchta R (2015) High morphological plasticity and global geographical distribution of the Pacific broad tapeworm *Adenocephalus pacificus* (syn. *Diphyllobothrium pacificum*): Molecular and morphological survey. Acta Trop 149: 168–178. doi: 10.1016/j.actatropica.2015.05.017 26001974 10.1016/j.actatropica.2015.05.017

[18] Maltsev VN, Gavrilova NA (1994) Variation of morphological characters within different parts of the strobila in cestodan genus *Diphyllobothrium* (Cestoda, Diphyllobothriidae). Zool Zh 1994: 59–62. (In Russian.)

[19] Kuchta R, Caira JN (2010) Three new species of *Echinobothrium* (Cestoda: Diphyllidea) from Indo-Pacific stingrays of the genus *Pastinachus* (Rajiformes: Dasyatidae). Folia Parasitol 57: 185–196. 20941910 10.14411/fp.2010.025

[20] Maejima J, Yazaki S, Fukumoto S (1983) Morphological comparison of eggs between marine species and freshwater species in diphyllobothriid cestodes. Jpn J Parasitol 32: 27–42. (In Japanese.)

[21] Chai JY, Murrell KD, Lymbery AJ (2005) Fish-borne parasitic zoonoses: Status and issues. Int J Parasitol 35: 1233–1254. 16143336 10.1016/j.ijpara.2005.07.013

[22] Kuchta R, Esteban J-G, Brabec J, Scholz T (2014) Misidentification of *Diphyllobothrium* species related to global fish trade, Europe. Emerg Inf Dis 20: 1955–1957. 10.3201/eid2011.140996 PMC4214320 25340877

[23] Kuhlow F (1953) Über die Entwicklung und Anatomie von *Diphyllobothrium dendriticum*, Nitzsch 1824. Z Parasitenkd 16: 1–35. 13137365 10.1007/BF00260407

[24] Torres P, Torres J, Garrido O, Thibaut J (1989) Investigaciones sobre Pseudophyllidea (Carus, 1813) en el sur de Chile. X. Observaciones experimentales sobre la coexistencia de plerocercoides de *Diphyllobothrium latum* (L.) y *Diphyllobothrium dendriticum* (Nitszch) en salmónidos de la cuenca del río Valdivia. Arch Med Vet 21: 51–57.

[25] Maejima J, Yazaki S, Fukumoto S (1991) Comparative studies on egg-sizes and -forms of various *Diphyllobothrium* species from man in Japan. Jpn J Parasitol 40: 170–176. (In Japanese.)

[26] Magath T (1919) The eggs of *Diphyllobothrium latum*. J Am Med Assoc 73: 85–87.

[27] Kim H-J, Eom KS, Seo M (2014) Three cases of *Diphyllobothrium nihonkaiense* infection in Korea. Kor J Parasitol 52: 673–676. 10.3347/kjp.2014.52.6.673 PMC4277031 25548420

[28] Yazaki S, Fukumoto S, Maejima J, Miyahara M (1990) Comparative observations of *Diphyllobothrium pacificum* from a man and from fur seals. J Yonago Med Ass 41: 204–210. (In Japanese.)

[29] Hilliard DK (1972) Studies on the helminth fauna of Alaska. LI. Observations on eggshell formation in some diphyllobothriid cestodes. Can J Zool 50: 585–592. 5063640 10.1139/z72-080

[30] Yamane Y, Seki R, Okada N (1976) Comparative observation on surface topography of teguments and egg shells of diphyllobothriid cestodes by scanning electron microscopy. Yonago Acta Med 20: 55–65. 1025934

[31] Wicht B, Yanagida T, Scholz T, Ito A, Jiménez JA, et al (2010) Multiplex PCR for differential identification of broad tapeworms (Cestoda: *Diphyllobothrium*) infecting humans. J Clin Microbiol 48: 3111–3116. doi: 10.1128/JCM.00445-10 20592146 10.1128/JCM.00445-10 PMC2937707

